# Disentangling Diversity Patterns in Sandy Beaches along Environmental Gradients

**DOI:** 10.1371/journal.pone.0040468

**Published:** 2012-07-06

**Authors:** Francisco R. Barboza, Julio Gómez, Diego Lercari, Omar Defeo

**Affiliations:** UNDECIMAR, Department of Ecology and Evolution, Faculty of Science, University of the Republic, Montevideo, Uruguay; McGill University, Canada

## Abstract

Species richness in sandy beaches is strongly affected by concurrent variations in morphodynamics and salinity. However, as in other ecosystems, different groups of species may exhibit contrasting patterns in response to these environmental variables, which would be obscured if only aggregate richness is considered. Deconstructing biodiversity, i.e. considering richness patterns separately for different groups of species according to their taxonomic affiliation, dispersal mode or mobility, could provide a more complete understanding about factors that drive species richness patterns. This study analyzed macroscale variations in species richness at 16 Uruguayan sandy beaches with different morphodynamics, distributed along the estuarine gradient generated by the Rio de la Plata over a 2 year period. Species richness estimates were deconstructed to discriminate among taxonomic groups, supralittoral and intertidal forms, and groups with different feeding habits and development modes. Species richness was lowest at intermediate salinities, increasing towards oceanic and inner estuarine conditions, mainly following the patterns shown for intertidal forms. Moreover, there was a differential tolerance to salinity changes according to the habitat occupied and development mode, which determines the degree of sensitivity of faunal groups to osmotic stress. Generalized (additive and linear) mixed models showed a clear increase of species richness towards dissipative beaches. All taxonomic categories exhibited the same trend, even though responses to grain size and beach slope were less marked for crustaceans and insects than for molluscs or polychaetes. However, supralittoral crustaceans exhibited the opposite trend. Feeding groups decreased from dissipative to reflective systems, deposit feeders being virtually absent in the latter. This deconstructive approach highlights the relevance of life history strategies in structuring communities, highlighting the relative importance that salinity and morphodynamic gradients have on macroscale diversity patterns in sandy beaches.

## Introduction

Sandy shores are dynamic environments defined by the interaction between wave energy, tides and wind regimes, which produce a continuum from microtidal reflective beaches (narrow, steep and with coarse sand) to macrotidal dissipative beaches (wide, flat and with fine sand) [1]. A main paradigm in sandy beach ecology argues that macrofaunal richness increases from reflective to dissipative beaches [2]–[9]. Two recent meta-analyses on data coming from four continents confirmed that species richness increases from microtidal reflective beaches to macrotidal dissipative beaches and from temperate to tropical regions [9], [10]. However, these studies considered the macrofauna in an aggregated way and did not differentiate between taxa or other groups of species [11].

Despite the general biodiversity patterns described for sandy beaches and the hypotheses originated to explain them, many of the causes responsible for these patterns remain unknown. Most studies carried out at the community level rarely consider the characteristics of the species' life cycle, their taxonomic affiliation, or the beach zone occupied by the different macrofaunal components. This is of importance, taking into account that aggregated variables, such as species richness, reduce to a single value the particularities of a set of species that result from the interaction of biotic and abiotic processes. This integration in a single variable is a problem in the elucidation of the mechanisms underlying biodiversity patterns, since several processes operating at multiple scales may govern spatial variations in diversity [12]–[14]. In the context of sandy beach ecology, a recent analysis of data from 63 beaches along 2 ecorregions in Southwestern Atlantic beaches, showed that macrofaunal community components exhibited contrasting responses between beach types [11]. Thus, it is essential to consider the characteristics of each group in order to understand the observed patterns and to disentangle the drivers of diversity according to beach morphodynamics.

In addition to the changes imposed by beach morphodynamics, salinity has been identified as a critical variable that affects biodiversity patterns [15]–[17]. Variations in salinity, rather than salinity itself, drive variations in abundance and distribution of macrofaunal components along estuarine gradients [18], [19]. In this context, differential susceptibility of species with different development modes to salinity variations could cloud the understanding of observed patterns. Indeed, in species with direct development (e.g. peracarids), internal incubation allowed them to overcome salinity variations [20], [21], while species with indirect development are affected to a greater extent by salinity gradients [22]. However, no attempts have been made to deconstruct these patterns, i.e. to evaluate separately the simultaneous effects generated by estuarine and morphodynamic gradients in various components of the macrofauna.

Uruguayan sandy beaches located along the estuarine gradient generated by the Rio de la Plata (RdlP), cover a wide morphodynamic range [3], providing an ideal framework for evaluating the simultaneous action of both factors in the patterns of species richness, both aggregated and discriminated by community component. In this paper we deconstruct macrofaunal patterns of species richness in 16 Uruguayan sandy beaches. We compare richness patterns among taxonomic and feeding groups, species with different development modes and semi-terrestrial versus intertidal forms, in order to better understand richness patterns in relation to variations in the physical environment.

## Materials and Methods

### Ethics statement

No specific ethical or institutional permits were required for the described field studies according to the Uruguayan Law N° 18.611, because invertebrate species are not protected by any legal regulation. All sampling was conducted outside protected and privately-owned areas. Field studies did not involve endangered or protected species. All efforts were made to ameliorate suffering of animals.

### Study area

**Figure 1:**
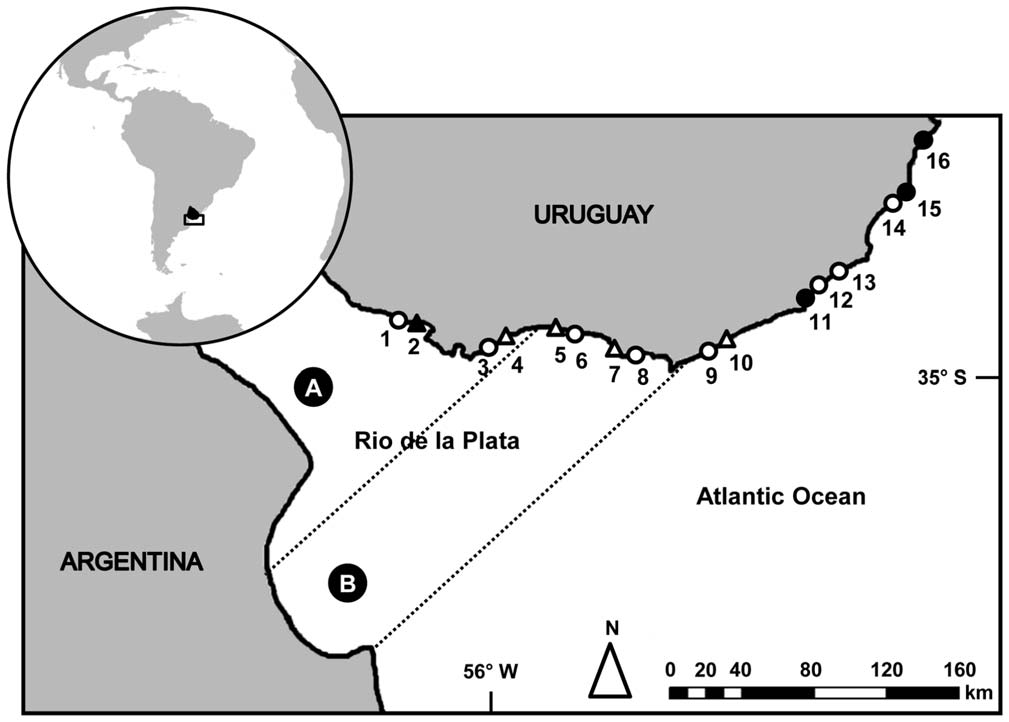
Location map of the 16 Uruguayan sandy beaches surveyed during 2 consecutive years. Beaches are numbered from west to east and categorized as reflective (○), intermediate (▵) and dissipative (•) based on Dean's parameter Ω. Beach 2 (▴) was not classified according to this criterion (see text for details). A and B indicate the inner and outer regions of the Rio de la Plata estuary.

The RdlP, located on the west coast of the South Atlantic Ocean, is the world's widest estuary (Fig. 1). Landward waters coming from Parana and Uruguay Rivers, in contact with the ocean water, generate a marked salinity gradient that extends along hundreds of km of the Uruguayan coast [23]. Salinity controls water density, while temperature is involved in the determination of horizontal gradients [24]. Winds are the main determinant of water level variations, because astronomic tides are <0.5 m.

### Sampling design

16 sampling sites located between the inner estuary (beach 1) and the fully marine area (beach 16) were selected considering, whenever possible, close pairs of beaches with the same salinity range but markedly different morphodynamic features [16] (Fig. 1). Each beach was sampled bimonthly form July 1999 to April 2001. During each sampling event, three transects were laid perpendicular to the shoreline and spaced 8 m apart, with sampling units beginning at the base of the dunes and continuing at 4 m intervals in a seaward direction, until the lower limit of the swash zone was reached. Sampling units at each transect were taken with a metal cylinder 27 cm in diameter and 40 cm deep. The sediment was sieved through a 0.5 mm mesh and the retained organisms were fixed (formalin 5%) for their classification at the species level (whenever possible).

Environmental information was obtained for each beach. At the swash zone, salinity and water temperature were measured with an YSI 33 thermosalinometer. Beach slope was also determined according to Emery [25], wave height was visually recorded and wave period was estimated with a stopwatch. Swash width was measured as the distance covered by the water on the beach face slope after a wave collapses on the sand, while beach width was determined as the distance between the base of the dunes and the lower limit of the swash zone. Sand compaction was measured at each sampling unit using a piston pocket penetrometer [26]. Sediments samples were collected for sedimentological analysis. A fraction was oven-dried, weighed, burned and finally weighed again to estimate sand moisture and organic matter content. Another sediment subsample was used for granulometric analysis [27].

### Data analysis

After completion of the matrix of physical factors, Dean's parameter [1] was calculated as follows:
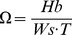
where *Hb* is breaker height (m), *Ws* is sand fall velocity (m·s^−1^) and *T* is the wave period (s). Ω<2 characterizes reflective beaches, Ω>5 defines dissipative ones and 2<Ω<5 characterizes intermediate beach states. Salinity range was defined as the difference between the lowest and highest salinity values measured in the swash zone of each beach during study period.

α - diversity (species richness) was estimated as the total number of species identified at each site throughout the sampling period. Thus, eventual fluctuations in species richness due to seasonality and intrinsic characteristics of the species were eliminated. Species richness was deconstructed using 4 different strategies for species grouping, as well as considering individual species, resulting in 5 strategies of analysis (see details in Defeo & McLachlan [11]): (1) taxonomy (molluscs, polychaetes, crustaceans and insects); (2) beach zone occupied (supralittoral and intertidal); (3) development mode (direct developers and species with indirect development and planktonic larvae); (4) feeding mode (scavengers/predators, deposit feeders and filter feeders); and (5) species level: effects of taxonomy, beach zone occupied, development mode and feeding mode at the species level were separated by evaluating the response of 4 species to salinity and grain size. In order to do so, abundance was expressed as individuals per strip transect (IST, ind·m^−1^).

Relationships between environmental variables (mean values for the whole period) and species richness were carried out using linear and nonlinear models. Best models were selected according to the coefficient of determination (R^2^) and statistical significance. In addition, a Generalized Additive Mixed Model (GAMM) was implemented using gamm4 R package [28], to determine the effect and the relative importance of environmental variables on total species richness. In this case, a categorical site variable was included as random intercept, reducing the number of estimated parameters [29] and developing a general model for the study area. A Poisson distribution and a log-link function were used for fitting. Potential predictors included in the model were chosen using information provided by bivariate analysis. Biological and physical information obtained in each sampling event was used, in order to consider the variation within each beach. Environmental predictors were initially included as smooth terms using penalized regression splines with 3 degrees of freedom [30], [31]. The Akaike's Information Criterion (AIC) was used to evaluate each model's fit and parsimony in the modelling process. Submodels were obtained by eliminating non-significant variables, until the model that included all significant variables with the lowest AIC was accomplished. In order to obtain a parametric version, model coefficients were estimated by a Generalized Linear Mixed Model (GLMM), using lme4 R package [32], substituting non-parametric functions by similar parametric ones. Final GAMM and GLMM estimates were compared by AIC, to determine if they had a similar empirical support (ΔAIC<2) [33].

## Results

### Environmental factors

An environmental characterization of the 16 sandy beaches included in this study is presented in Table 1. Considering mean values, salinity increased from west to east and varied between 4.40 (beach 1) and 29.20 (beach 16). Water temperature was highest at beach 2 (21.20°C) and lowest at beach 4 (16.40°C). Slope and grain size were highest (8.82% and 0.66 mm, respectively) at beach 8, and lowest at beaches 2 (1.42%) and 4 (0.15 mm). Wave period tended to 0.00 s at beach 2 and was highest at beach 11 (7.79 s). Swash width was lowest at beach 2 (1.27 m) and highest at beach 16 (12.73 m). Sand compaction varied between 4.60 kg·cm^−2^ (beach 11) and 2.20 kg·cm^−2^ (beach 9) and sand moisture between 19.9% (beach 2) and 5.2% (beach 3). Dean's parameter Ω varied between 1.3 (beach 3) and 6.3 (beach 16), including in this way reflective, intermediate and dissipative beaches. Due to the fact that the wave period registered at beach 2 during the two years of sampling was virtually 0, Dean's parameter was not estimated for this beach.

**Table 1:** Environmental characterization of the 16 beaches sampled along the Rio de la Plata estuary and the oceanic Uruguayan coast.

Variable	Mean value		Range	
	Minimum	Maximum	Minimum	Maximum
Salinity	4.40 (1)	29.20 (16)	0.10	34.30
Water temperature (°C)	16.40 (4)	21.20 (2)	7.40	32.00
Slope (%)	1.42 (2)	8.82 (8)	0.72	14.96
Grain size (mm)	0.15 (4)	0.66 (8)	0.13	0.95
Wave period (s)	0.00 (2)	7.79 (11)	0.00	13.64
Swash width (m)	1.27 (2)	12.73 (16)	0.00	20.00
Compaction (kg·cm^−2^)	2.20 (9)	4.60 (11)	1.00	5.00
Sand moisture (%)	5.2 (3)	19.9 (2)	2.45	23.94
Dean's parameter	1.3 (3)	6.3 (16)	0.41	10.24

For mean values, the respective beaches are indicated in parenthesis following the numeration presented in Figure 1.

### Integrated patterns of species richness

**Figure 2:**
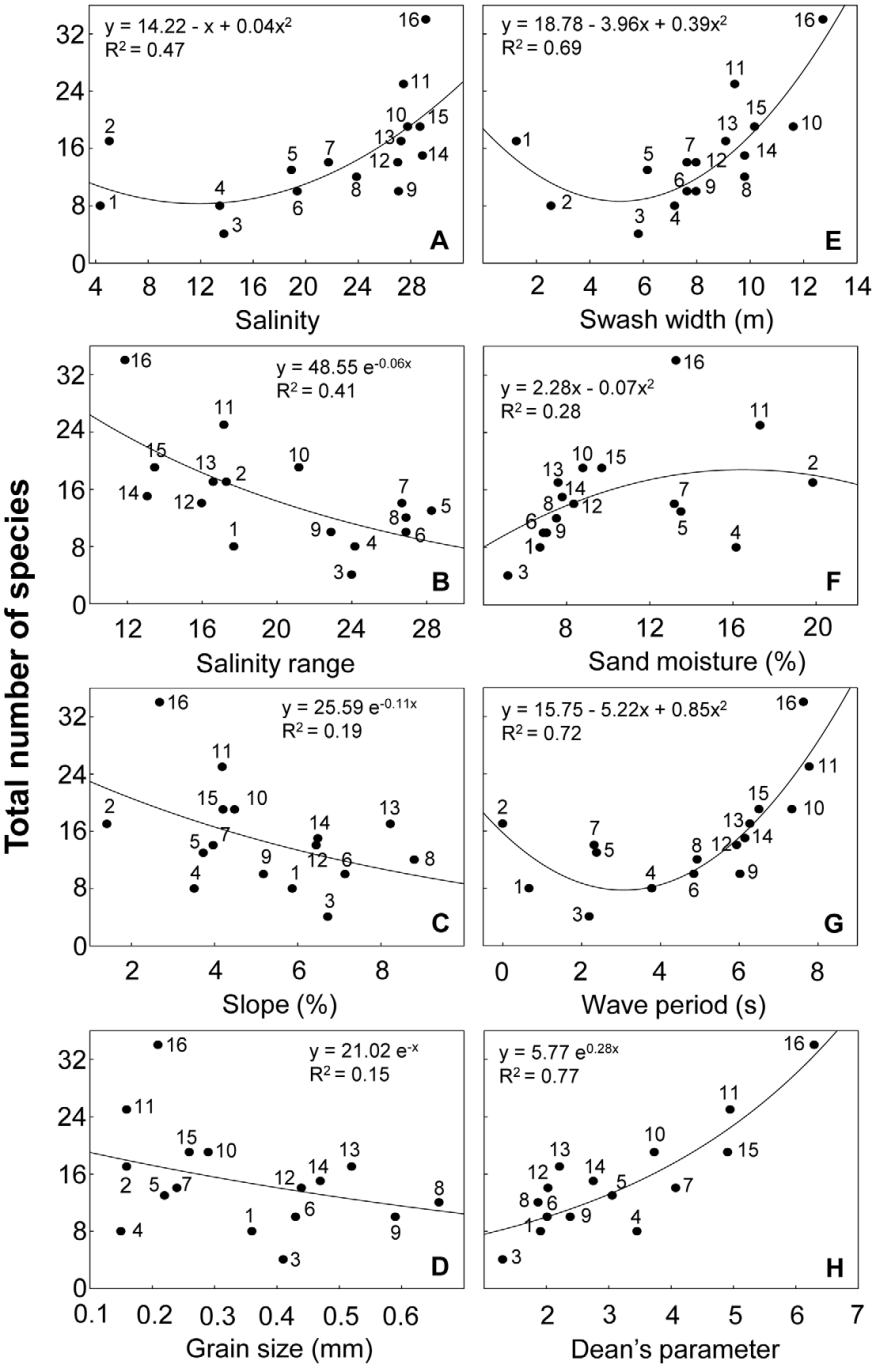
Models relating species richness and environmental variables in Uruguayan sandy beaches. Relationship between species richness and: (A) salinity, (B) salinity range, (C) slope, (D) grain size, (E) swash width, (F) sand moisture, (G) wave period and (H) Dean's parameter Ω. Beaches are numbered following Figure 1. Beach 2 was not included in H (see text for details). Statistical details are presented in Table S1.

Large scale variation in species richness was related to environmental variables through bivariate models (Table S1). Species richness: 1) was lowest at intermediate salinity values (between 10 and 14), increasing towards fully marine and freshwater conditions (Fig. 2A); 2) decreased with salinity range, slope and grain size (Fig. 2B–D), exhibiting their lowest values at intermediate swash widths (Fig. 2E) and wave periods (Fig. 2G), and their highest values at intermediate sand moistures (Fig. 2F); and 3) exponentially increased with Dean's parameter Ω (Fig. 2H). These trends clearly denoted an increase of this biological descriptor from reflective to dissipative beaches.

**Figure 3:**
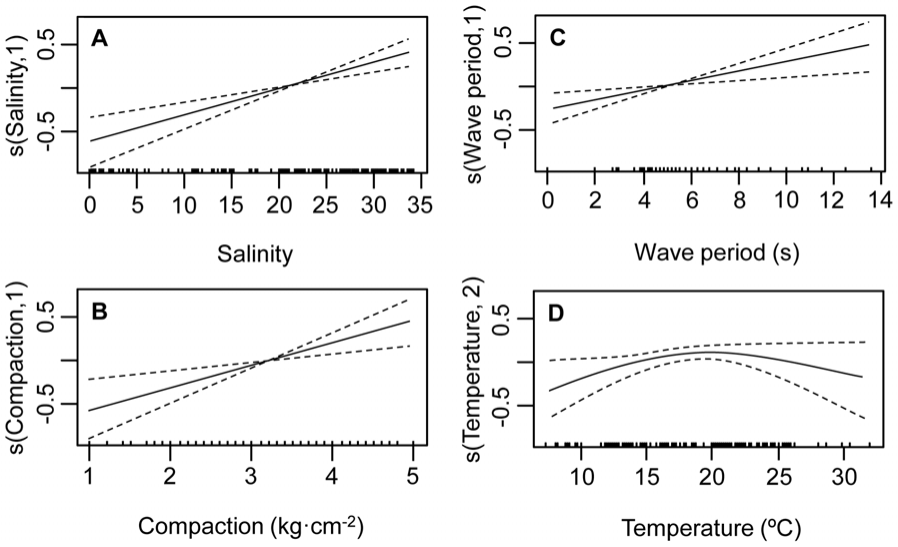
Results of the Generalized Additive Mixed Models relating species richness and environmental predictors. Partial effects of environmental predictors on species richness are shown (solid line). Dotted lines indicate 2 times the standard error. The marks on the x-axis show the distribution of measured values for each predictor.

The results of the modelling process are shown in Table 2. The most parsimonious GAMM reached an AIC of 174.6, differing from the previous one in 4.5 units, thus indicating strong empirical support when compared with other models. This final GAMM only retained 4 of the original 9 variables included in the complete model (Fig. 3). The relative importance of these variables were, in decreasing order: salinity, sand compaction and wave period, which were retained as linear predictors (Fig. 3A–C), and water temperature, included in the model as a nonlinear predictor with 2 degrees of freedom (Fig. 3D). Species richness increased with salinity, sand compaction and wave period and followed a negative quadratic shape in relation to temperature, with an optimum at 20°C. The parametric model (GLMM; Tables 2 and S2) had an AIC that differs in 0.5 in relation to the more parsimonious GAMM, having essentially the same empirical support.

**Table 2:** Model building results for the Generalized Additive Mixed Models (GAMM) and Generalized Linear Mixed Models (GLMM) relating species richness and environmental predictors in the Uruguayan coast.

Model type	Model expression	AIC
GAMMs	s(Salinity, 3)+s(Sand compaction, 3)+s(Water temperature, 3)+s(Wave period, 3)+s(Slope, 3)+s(Wave height, 3)+s(Grain size, 3)+s(Sand moisture, 3)+s(Swash width, 3)	205.2
	s(Salinity, 2)+s(Wave period, 2)+s(Sand compaction, 2)+s(Water temperature, 2)+s(Slope, 2)+s(Wave height, 2)+s(Sand moisture, 2)+s(Grain size, 2)+s(Swash width, 2)	190.7
	s(Salinity, 2)+s(Wave period, 2)+s(Sand compaction, 2)+s(Water temperature, 2)+s(Slope, 2)+s(Wave height, 2)+s(Sand moisture, 2)+s(Grain size, 2)	186.8
	s(Salinity, 2)+s(Wave period, 2)+s(Sand compaction, 2)+s(Water temperature, 2)+s(Slope, 2)+s(Wave height, 2)+s(Sand moisture, 2)	183.2
	s(Salinity, 2)+s(Sand compaction, 2)+s(Wave period, 2)+s(Water temperature, 2)+s(Slope, 2)+s(Wave height, 2)	180.1
	s(Salinity, 2)+s(Wave period, 2)+s(Sand compaction, 2)+s(Water temperature, 2)+s(Slope, 2)	180.0
	s(Salinity, 2)+s(Sand compaction, 2)+s(Wave period, 2)+s(Water temperature, 2)	179.1
	Salinity+Sand compaction+Wave period+s(Water temperature, 2)	174.6
GLMM	Salinity+Sand compaction+Wave period+poly(Water temperature, 2)	174.1

Main models obtained from the modelling process are expressed in R language. Numbers presented in the model expression refer to the degrees of freedom used in smooth terms (s). The respective Akaike's Information Criterion (AIC) values are presented. All the terms of the last GAMM were significant (p<0.05). The estimated GLMM coefficients are prese.

### Taxonomic deconstruction

**Figure 4:**
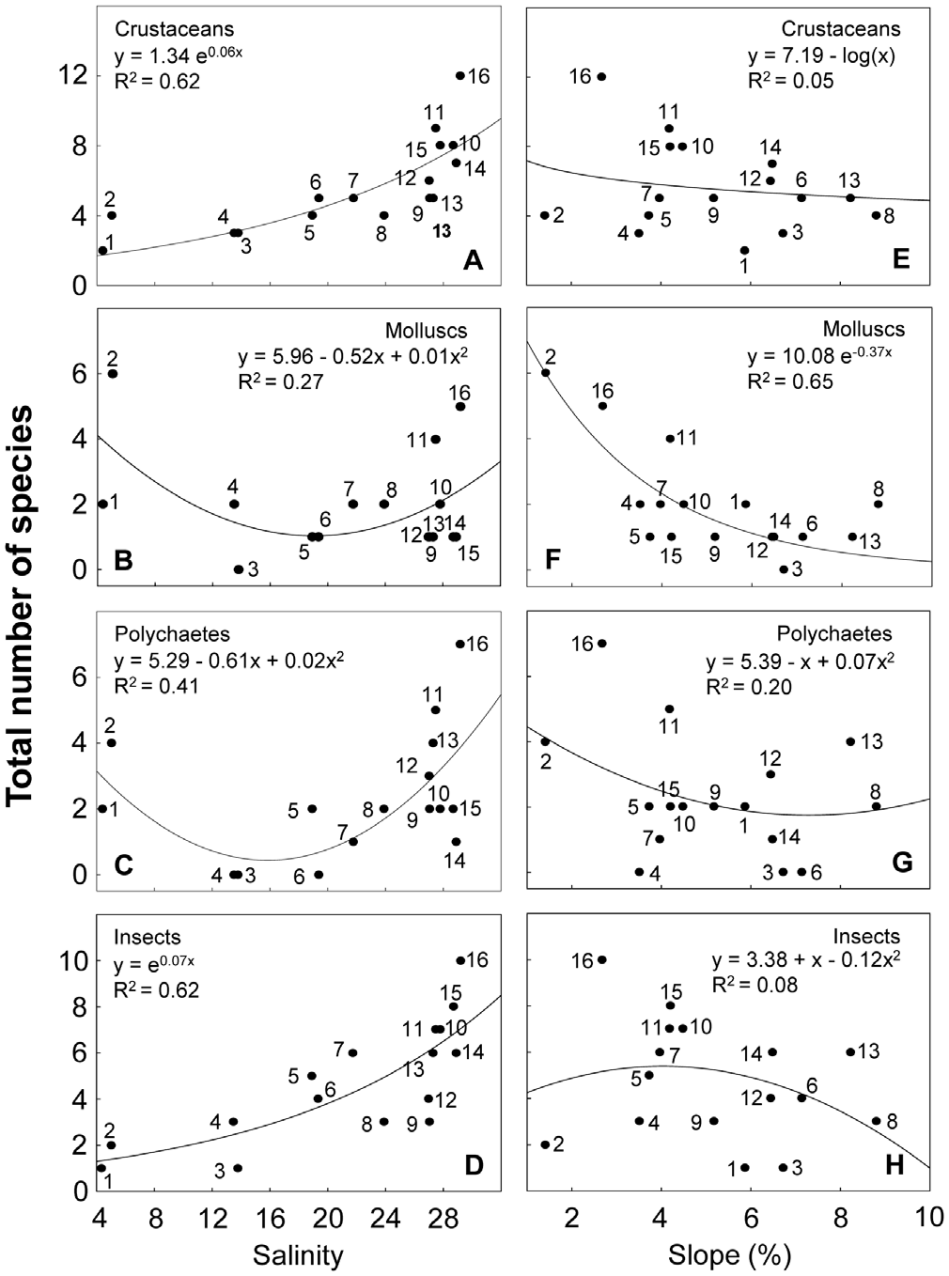
Relationship between species richness discriminated by taxonomic group and salinity and slope. Beaches are numbered following Figure 1. Statistical details of the models fitted are presented in Table S3.

**Figure 5:**
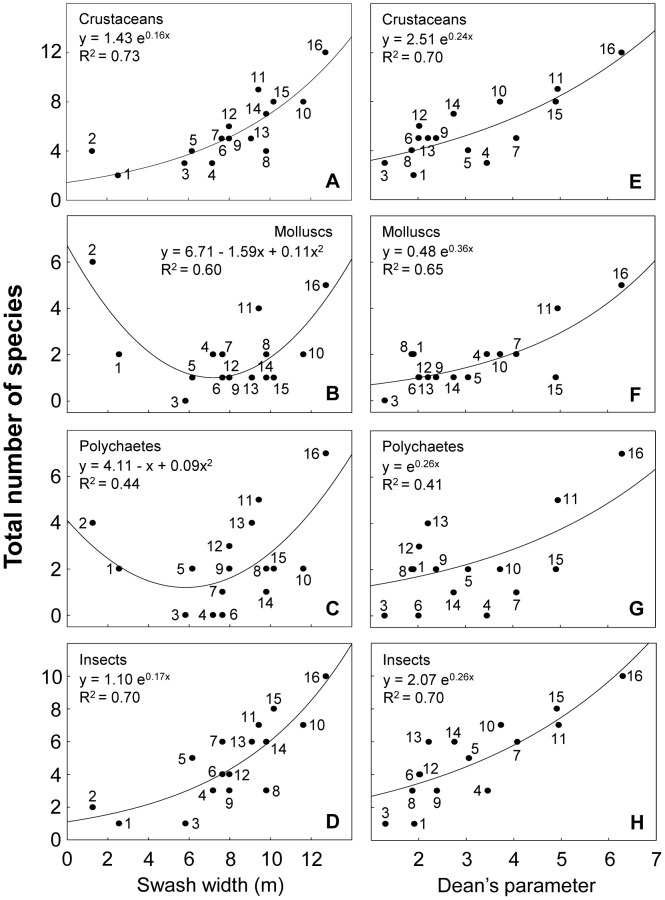
Relationship between species richness discriminated by taxonomic group and swash width and Dean's parameter Ω. Beaches are numbered following Figure 1. Beach 2 was not included in E, F, G and H (see text for details). Statistical details of the models fitted are presented in Table S3.

The benthic macrofauna included representatives of crustaceans, molluscs and polychaetes (72%), as well as insects (23%). Deconstruction based on taxonomy showed a wide range of responses to environmental variables (Table S3). Concerning salinity, crustaceans and insects increased towards fully marine conditions (Fig. 4A, D), whereas molluscs and polychaetes showed their lowest richness at intermediate salinities, increasing towards freshwater and oceanic beaches (Fig. 4B, C). Crustaceans, molluscs and polychaetes decreased with salinity range, while insects exhibited their lowest richness at intermediate values (Fig. S1). Crustaceans and molluscs decreased with beach slope (Fig. 4E, F), while quadratic models were obtained for polychaetes and insects (Fig. 4G, H). Nevertheless, for crustaceans and insects, the best models explained a very small portion of the variance (Table S3). All faunal categories responded in relation to grain size in the same way described for beach slope (Fig. S1). Crustaceans and insects increased with swash width and wave period (Fig. 5A, D; Fig. S2), unlike molluscs and polychaetes, which presented their lowest richness at intermediate values (Fig. 5B, C; Fig. S2). Molluscs increased with sand moisture, whereas crustaceans, polychaetes and insects showed highest species richness at intermediate values (Fig. S2). The four taxonomic groups exponentially increased with Dean's parameter Ω, explaining in all cases more than 40% of the variance (Fig. 5E–H).

### Deconstruction by beach zone

**Figure 6:**
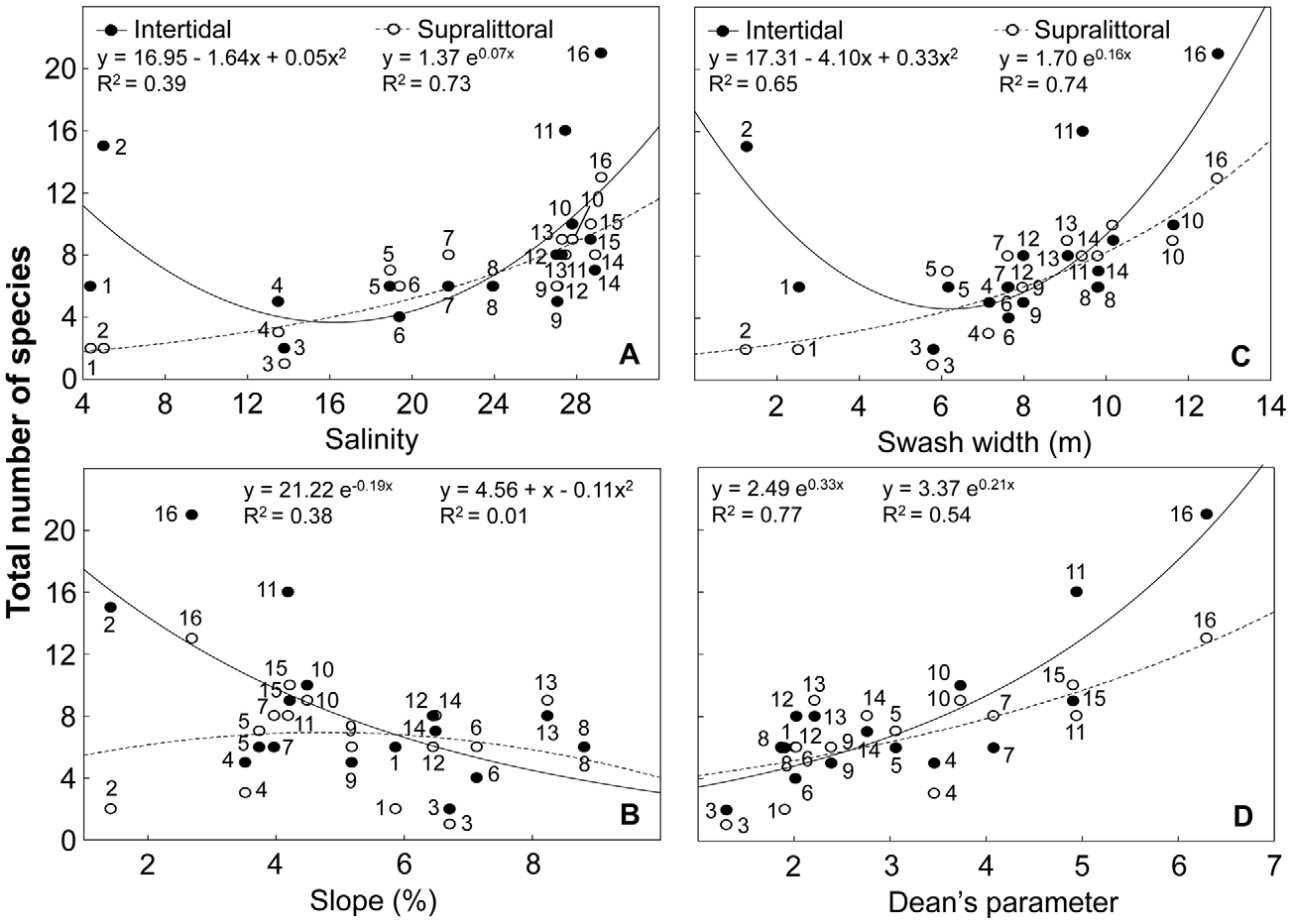
Relationship between species richness discriminated by beach zone occupied and environmental variables. (A) salinity, (B) slope, (C) swash width and (D) Dean's parameter Ω. Beaches are numbered following Figure 1. Beach 2 was not included in D (see text for details). Statistical details of the models fitted are presented in Table S3. Crustaceans (2 species) and insects were included in the supralittoral category.

Species richness of intertidal and supralittoral forms responded in different ways in relation to several environmental variables (Table S3). Intertidal species exhibited their lowest richness at intermediate salinities, swash widths and wave periods, decreasing with salinity range, beach slope and grain size, and increasing with sand moisture (Fig. 6 and Fig. S3). Supralittoral species increased with salinity, swash width and wave period, reaching lowest values at intermediate salinity ranges, and their highest at intermediate sand moistures. These species showed a weak response to beach slope and grain size (Fig. 6 and Fig. S3). Both faunal categories increased with Dean's parameter Ω (Fig. 6).

### Deconstruction by development mode

**Figure 7:**
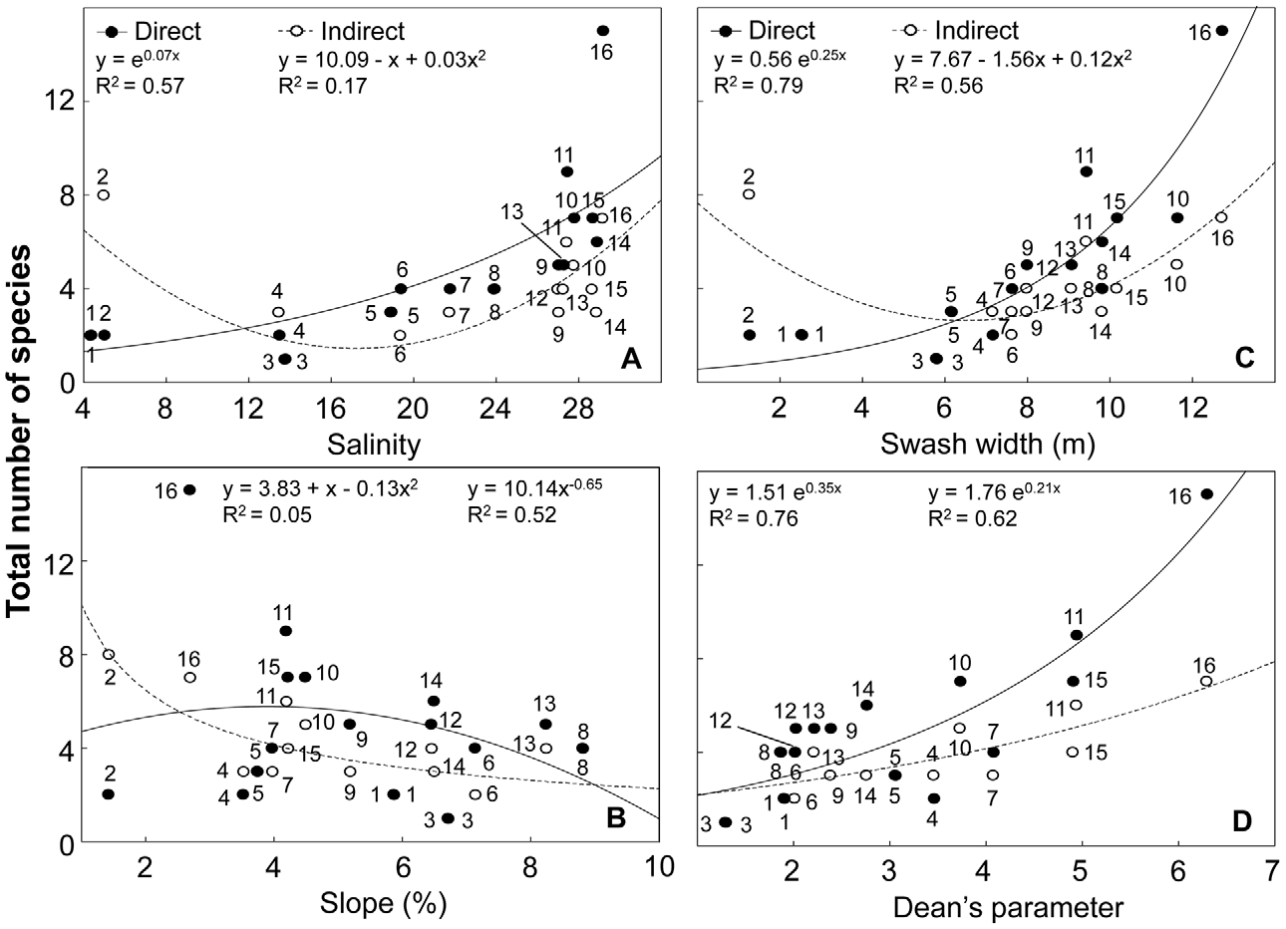
Relationship between species richness discriminated by development mode and environmental variables. (A) salinity, (B) slope, (C) swash width and (D) Dean's parameter Ω. Beaches are numbered following Figure 1. Beach 2 was not included in D (see text for details). Statistical details of the models fitted are presented in Table S3.

Categories defined by development mode exhibited contrasting responses to environmental variables (Table S3). Direct developers increased with salinity, swash width and wave period, decreased with salinity range and showed their highest richness at intermediate sand moistures (Fig. 7; Fig. S4). This group was barely associated to variations in slope and grain size. Indirect developers showed their lowest richness at intermediate salinities, swash widths and wave periods, decreased with salinity range, beach slope and grain size, and increased with sand moisture (Fig. 7; Fig. S4). Finally, the species richness of direct and indirect developers increased exponentially with Dean's parameter Ω (Fig. 7).

### Deconstruction by feeding mode

**Figure 8:**
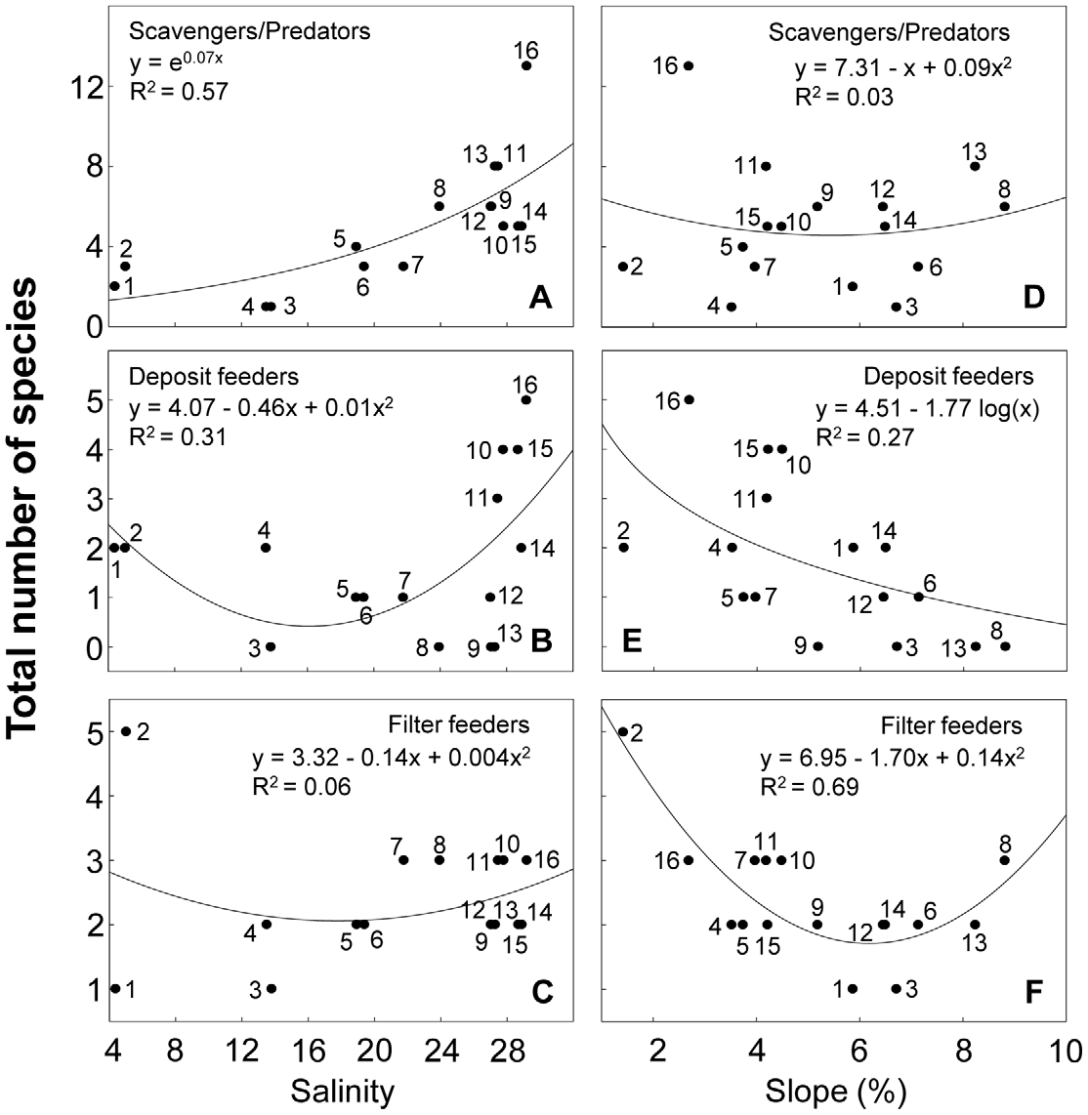
Relationship between species richness discriminated by feeding mode and salinity and slope. Beaches are numbered following Figure 1. Statistical details of the models fitted are presented in Table S3.

**Figure 9:**
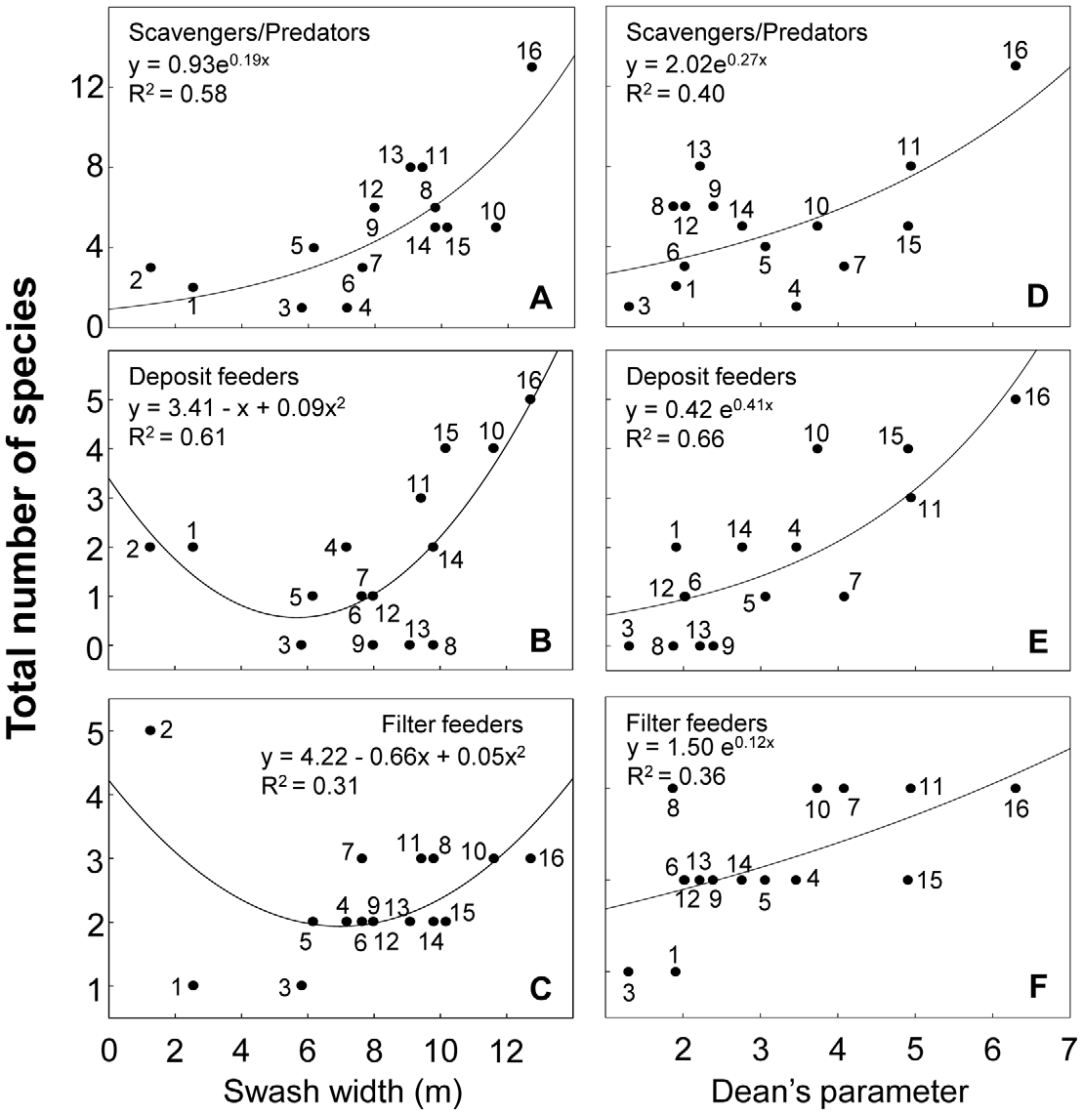
Relationship between species richness discriminated by feeding mode and swash width and Dean's parameter Ω. Beaches are numbered following Figure 1. Beach 2 was not included in D, E and F (see text for details). Statistical details of the models fitted are presented in Table S3.

Scavengers/predators increased with salinity, while filter and deposit feeders exhibited their lowest richness at intermediate salinities (Fig. 8A–C). The three feeding groups decreased with salinity range (Fig. S5). Scavengers/predators and filter feeders showed their lowest richness at intermediate slopes and grain sizes, while deposit feeders showed decreasing patterns (Fig. 8D–F and Fig. S5). Scavengers/predators increased with swash width, whereas deposit and filter feeders displayed their lowest richness at intermediate swash widths (Figs. 9A–C). The same patterns were observed in relation to wave period (Fig. S6). Scavengers/predators and deposit feeders showed their highest richness values at intermediate sand moistures, whereas filter feeders increased with sand moisture (Fig. S6). All categories exponentially increased with Dean's parameter (Fig. 9D–F). Statistical details are summarized in Table S3.

### Deconstruction by species

**Figure 10:**
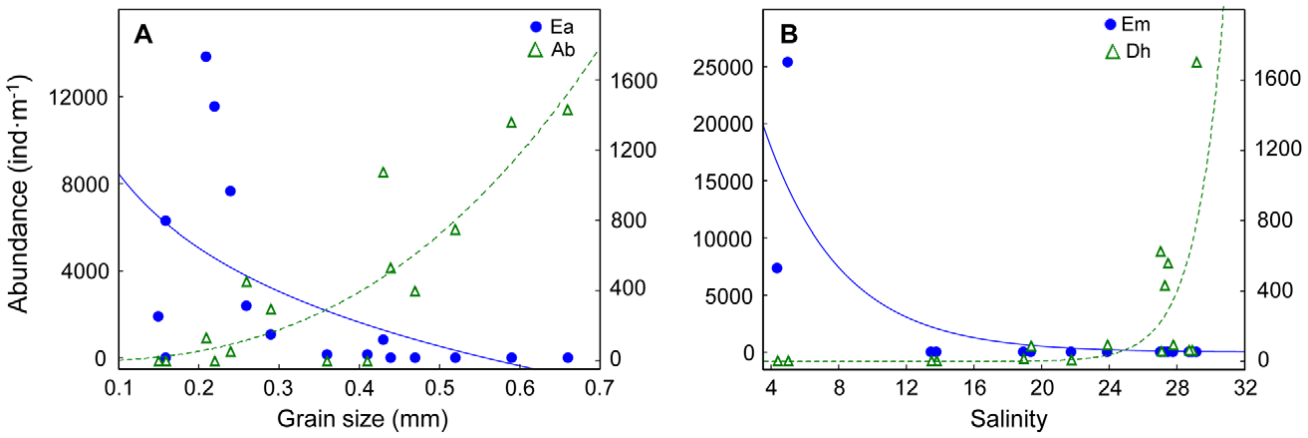
Relationship between abundance of selected sandy beach species and grain size (A) and salinity (B). Beaches are numbered following Figure 1. The y-axis on the left corresponds to the species represented in blue, while the right to species represented in green. Ea: *Excirolana armata*, Ab: *Altantorchestoidea brasiliensis*, Em: *Erodona mactroides*, Dh: *Donax haleyanus*. Statistical details of the models fitted are presented in Table S4.

Abundance of *Atlantorchestoidea brasiliensis* (crustacean, supralittoral, direct developer, scavenger/predator) increased with grain size, while *Excirolana armata* (crustacean, intertidal, direct developer, scavenger/predator) abundance followed the opposite pattern (Fig. 10A). These contrasting responses to morphodynamics between sandy beach peracarid crustaceans could be mainly ascribed to the beach zone occupied, because both species have the same taxonomic affiliation, development and feeding modes. Concerning salinity, the bivalve *Erodona mactroides* (intertidal, indirect developer, filter feeder) only occurred in brackish environments, whereas the wedge clam *Donax hanleyanus* (intertidal, indirect developer, filter feeder) only occurred in fully marine conditions (Fig. 10B). This clearly denotes that contrasting patterns can be found at the species level in sandy beaches, even under identical characteristics in life history traits. Statistical details are summarized in Table S4.

## Discussion

### Integrated patterns of species richness

Macroscale distribution of the macrofauna in Uruguayan sandy beaches is determined by estuarine and morphodynamic gradients. Species richness attained its lowest values at estuarine beaches, increasing both to fully freshwater and oceanic conditions, in agreement with findings documented by Remane [34], Atrill [19] and Lercari & Defeo [16]. Species richness clearly decreased with salinity range, reinforcing the predictions of Atrill's linear model [19]. The reduced species richness in outer estuary beaches with high salinity ranges, suggests that sandy beach macrofauna has relatively low tolerance to large salinity variations. Both salinity and salinity range are recognized as key factors in structuring macrofaunal communities [16], [21], [22].

Our results are conclusive about the importance of morphodynamic features in explaining large scale distribution patterns of sandy beach biodiversity. The patterns obtained in relation to Dean's parameter Ω, slope and grain size, showed an increase in species richness from reflective to dissipative beaches, in line with worldwide findings [2]–[9]. In agreement with recent explanations about the control of biological patterns in sandy beaches, both swash climate and sand properties play a crucial role [10], [35], [36]. In this context, dissipative beaches, with benign swash climates (low turbulence, slow drainage and short swash periods) and fine sand, exhibit a greater number of species than reflective ones [10]. Spatial variations of species richness were also explained by variations in swash width and wave period, having a similar response to that shown for salinity. An increase in swash width and wave period from estuarine to oceanic beaches was also observed, highlighting the significant effect of the RdlP discharge on the hydrodynamics of the system, which in turn affects biodiversity [16]. Sand moisture was another relevant variable that explained spatial patterns in species richness, the quadratic model fitted between species richness and this variable being indicative of the existence of an optimal sand moisture value in which species richness is maximized. Beach 2 behaved as a tidal flat, having the highest sand moisture and the lowest slope [1]. These features, coupled with the freshwater conditions registered in this beach, particularly favour the establishment of intertidal species (e.g. *Erodona mactroides*; Fig. 10B) that do not usually occur in other sandy beaches, explaining the increase of species richness in the RdlP inner estuary.

Salinity, sand compaction and wave period were included in the most parsimonious GAMM and GLMM models, highlighting the role that estuarine and morphodynamic gradients have on large scale patterns of sandy beach biodiversity. The observed trends in relation to sand compaction and wave period evidence a clear increase in species richness towards dissipative beaches. Temperature was included as a main predictor of species richness, and the 20°C optimum observed coincides with the marked seasonal migration of several subtidal species (notably gastropods) to the intertidal fringe in summer and early autumn. In addition, this is the recruitment period of many invertebrates inhabiting sandy beaches in Uruguay, Argentina and Southern Brazil [22], [37]–[39].

### Taxonomic deconstruction

Crustaceans and insects increased with salinity, whereas molluscs and polychaetes reached their lowest richness at intermediate salinity values. This suggests a higher tolerance of crustaceans and insects to intermediate salinities. Some crustacean groups have adaptations that allow them to persist in environments with sharp salinity changes. Peracarids (e.g. *Excirolana armata*) are characterized by a prolonged parental care (i.e. internal brooding), which has been recognized as an evolutionary advantage for the protection of their offspring against osmotic stress [20], [21], [40], [41]. Thus, the persistence and dominance of crustaceans on beaches with intermediate salinities and large salinity ranges could be mainly attributed to the occurrence of peracarids. Despite being a group linked to the intertidal realm of the littoral active zone [42], [43], insects preferentially inhabit the transition zone with the terrestrial environment [44], being less affected by salinity variability. In this sense, the quadratic model exhibited between salinity and insect species richness evidences a less pronounced effect of salinity changes on this group.

Molluscs increased from reflective to dissipative beaches. Crustaceans followed the same trend, but were less affected by grain size and slope. These patterns could be explained by two reasons: 1) several intertidal crustaceans are tolerant to coarse sands and swash energy, due to their high mobility and superb burrowing abilities; and 2) a reduced pool of supralittoral crustaceans is more abundant in reflective beaches than in dissipative ones, being adapted to coarse sands and steep slopes [11]. Insects increased towards dissipative conditions, although they exhibited weak responses in relation to slope and grain size. However, insect richness could be underestimated because of their landward distribution and partial coverage by the sampling design (the dune zone was not fully sampled). Moreover, the sampling strategy (quadrat sampling during the day) could affect the detection of this group [45], [46] because insects are highly mobile and active during the night. Insects particularly occurred at beaches with large swash widths, because they forage at upper swash levels of high-energy beaches. Thus, habitat and resource quality and availability have ecological importance for this group.

### Deconstruction by beach zone

Supralittoral species dominate over intertidal species at intermediate salinities, showing a greater resistance of this group to osmotic stress. Moreover, intertidal species exhibited a decreasing trend with salinity range, suggesting a lower tolerance of this faunal category to salinity changes [22]. The quadratic pattern obtained for supralittoral species with salinity range could result from the relative independence on the aquatic environment of these organisms.

Intertidal species increased from reflective to dissipative beaches. These trends are in agreement with the predictions of the Swash Exclusion Hypothesis (SEH), which state that the harsh swash climate of reflective beaches [35], [36]: 1) increases the risk of removal of organisms from the swash zone and the probability of stranding of the organisms above the effluent line; and 2) reduces the feeding time due to the frequent swashes. This is also consistent with the results obtained at the population level, confirming the Habitat Harshness Hypothesis (HHH) [47], [48]. Supralittoral species also increased towards dissipative beaches (see Fig. 6D), and this pattern could be mainly explained by insects' dominance within this group. However, some supralittoral crustaceans responded in the opposite way [3], [38], [49]–[53]. The amphipod *Atlantorchestoidea brasiliensis* exhibited an increase in its abundance towards reflective beaches (Fig. 10A). This result gives support to the Habitat Safety Hypothesis (HSH) [54], which states that the combination of short swashes and steep slopes in reflective beaches mitigate water intrusion, protecting species adapted to live in the supralittoral zone.

### Deconstruction by development mode

Species with indirect development decreased at intermediate salinities, highlighting the sensitivity of these species to osmotic stress [22]. These species, characterized by free living larval stages, are exposed to changing environmental conditions throughout its life cycle, being particularly susceptible at early development stages [55]. This may explain the absence of species with indirect development in outer estuarine beaches of the RdlP, which show significant variability in salinity, even in the short-term [16]. In contrast, species with direct development (and parental care) were less affected by the osmotic stress in early life cycle stages, allowing them to settle and persist in systems with large salinity variations [21]. Species richness of both faunal categories increased towards dissipative conditions, exhibiting clear positive trends in relation to Dean's parameter Ω. The slight relationship between direct developers and grain size and beach slope, could be possibly linked to the great number of intertidal and supralittoral crustaceans that belong to this category.

### Deconstruction by feeding mode

Scavengers/predators were particularly resistant to salinity variations, as evidenced by its dominance in the outer estuary of the RdlP. This is strongly linked to their development mode, because many of these species have direct development (see previous section) and thus are particularly resistant to osmotic stress. Deposit feeders, integrated by species with both direct and indirect development, decreased in environments with large salinity variations, mainly due to the fact that species with free living larval forms almost disappeared in the outer estuarine zone of the RdlP.

All feeding modes increased towards dissipative conditions (see Fig. 9D–F). Nevertheless, differences among groups were observed. Scavengers/predators occurred in all beach types. However, scavenger crustaceans were particularly well-represented in reflective beaches, where deposit feeders were totally absent. In these systems, the high drainage rates in coarse grain precludes the accumulation of organic matter in the sediment, therefore preventing the establishment of these species [11], [55]. Filter feeders, entirely intertidal, require frequent submersions, and this may explain their increase in systems with high values of sand moisture [55]. In this context, another interpretation is the consideration of sand moisture as a proxy of better swash conditions for filter feeders. By contrast, scavengers/predators decreased in beach 2, a brackish system that behaves as a tidal flat with high sand moisture. The absence of supralittoral crustaceans because of habitat unsuitability (i.e. lethal salinities and water saturated sediments) could explain the observed patterns [16], [55].

**Figure 11:**
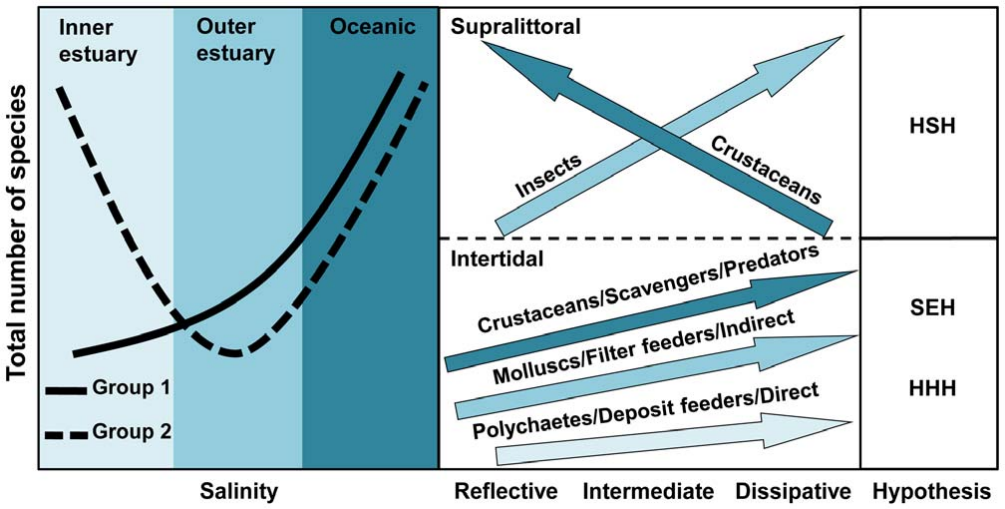
Conceptual model of the main responses of faunal categories to estuarine and morphodynamic gradients in sandy beaches. The models and hypotheses developed for estuarine and beach systems are included (see text for details). Group 1: Crustaceans, Insects, Supralittoral, Direct developers, Scavengers, Predators; Group 2: Molluscs, Polychaetes, Intertidal, Indirect developers, Filter feeders, Deposit feeders. HSH: Habitat Safety Hypothesis, SEH: Swash Exclusion Hypothesis, HHH: Habitat Harshness Hypothesis.

In summary, the deconstruction approach implemented in this study showed the crucial importance that species' life history traits have in our understanding of biodiversity patterns in sandy beaches. Thus, even though estuarine and morphodynamic gradients are relevant in the determination of macrofaunal community structure, the characteristics of different faunal groups are responsible for the pronounced differences observed in relation to these environmental gradients. Finally, our results give support and extend the theoretical framework developed for both sandy beach and estuarine systems (Fig. 11).

## Supporting Information

Figure S1 **Relationship between species richness discriminated by taxonomic group and salinity range and grain size.** Beaches are numbered following Figure 1. Statistical details of the models fitted are presented in Table S3.
(TIF)

Figure S2 **Relationship between species richness discriminated by taxonomic group and** sand moisture and wave period. Beaches are numbered following Figure 1. Statistical details of the models fitted are presented in Table S3.
(TIF)

Figure S3 **Relationship between species richness discriminated by beach zone occupied and environmental variables.** (A) salinity range, (B) grain size, (C) sand moisture and (D) wave period. Beaches are numbered following Figure 1. Statistical details of the models fitted are presented in Table S3.
(TIF)

Figure S4 **Relationship between species richness discriminated by development mode and environmental variables.** (A) salinity range, (B) grain size, (C) sand moisture and (D) wave period. Beaches are numbered following Figure 1. Statistical details of the models fitted are presented in Table S3.
(TIF)

Figure S5 **Relationship between species richness discriminated by feeding mode and salinity range and grain size.** Beaches are numbered following Figure 1. Statistical details of the models fitted are presented in Table S3.
(TIF)

Figure S6 **Relationship between species richness discriminated by feeding mode and sand moisture and wave period.** Beaches are numbered following Figure 1. Statistical details of the models fitted are presented in Table S3.
(TIF)

Table S1 **Best models relating species richness of the whole community and environmental variables.** ***p<0.001.
(DOC)

Table S2 **Fixed effects of the Generalized Linear Mixed Models relating species richness and environmental predictors.** Model components are expressed in R language. All terms were significant (p<0.05), with the exception of intercept and poly (Temperature, 2)1. SE: Standard Error.
(DOC)

Table S3 **Best models fitted for each deconstruction criterion based on taxonomic affiliation, beach zone occupied, development mode and feeding mode.** **p<0.01, ***p<0.001, n.s.: non-significant.
(DOC)

Table S4 **Best models relating abundance of selected species with grain size and salinity.** **p<0.01, ***p<0.001.
(DOC)
