# Supplementary figures and images for: Disentangling Diversity Patterns in Sandy Beaches along Environmental Gradients
Source: PLoS One. 2012 Jul 6;7(7):e40468. doi: 10.1371/journal.pone.0040468 (PMC3391285; doi:10.1371/journal.pone.0040468)

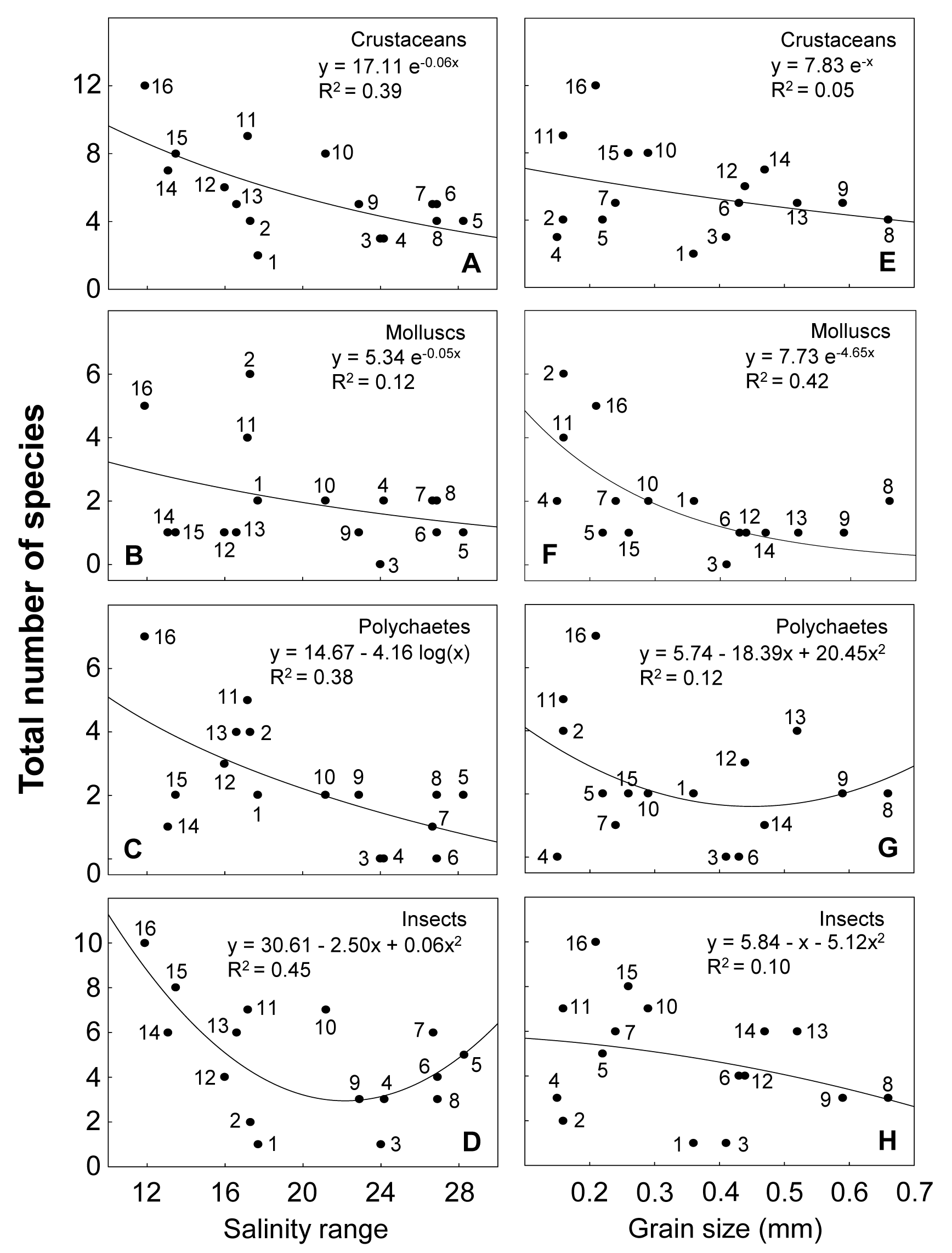

Supplement: Figure S1 — Relationship between species richness discriminated by taxonomic group and salinity range and grain size. Beaches are numbered following Figure 1. Statistical details of the models fitted are presented in Table S3. (TIF) [file pone.0040468.s001.tif]

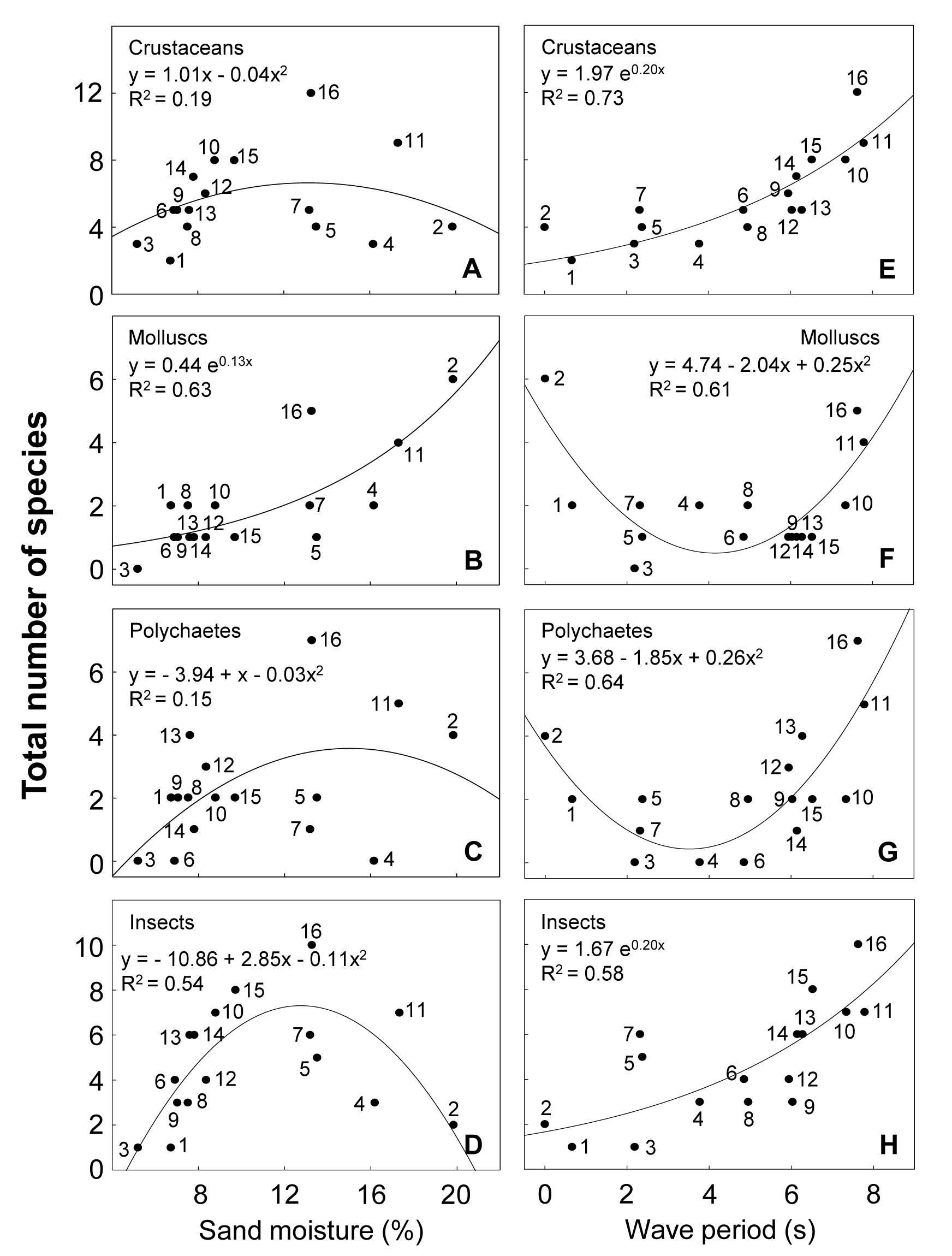

Supplement: Figure S2 — Relationship between species richness discriminated by taxonomic group and sand moisture and wave period. Beaches are numbered following Figure 1. Statistical details of the models fitted are presented in Table S3. (TIF) [file pone.0040468.s002.tif]

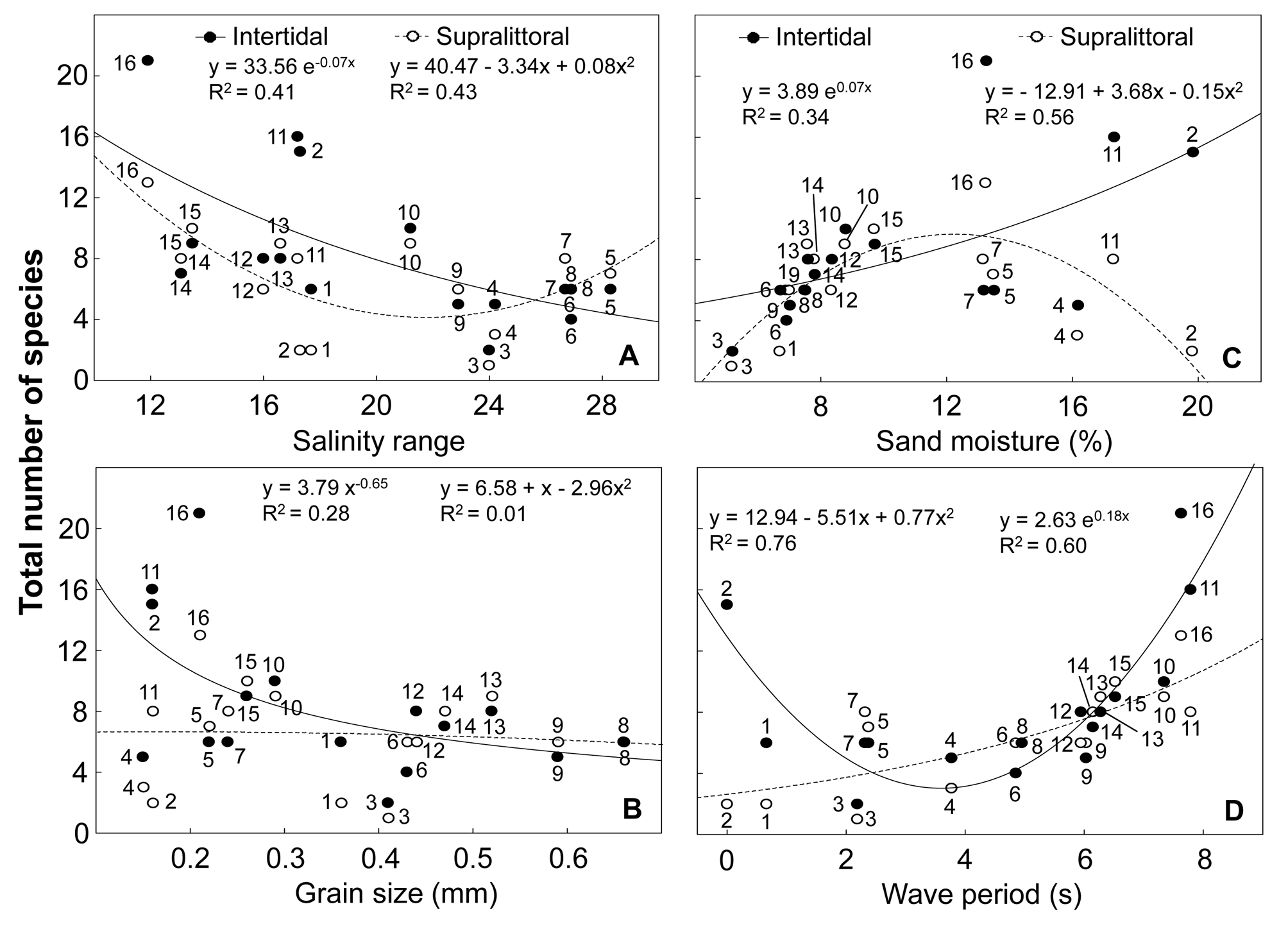

Supplement: Figure S3 — Relationship between species richness discriminated by beach zone occupied and environmental variables. (A) salinity range, (B) grain size, (C) sand moisture and (D) wave period. Beaches are numbered following Figure 1. Statistical details of the models fitted are presented in Table S3. (TIF) [file pone.0040468.s003.tif]

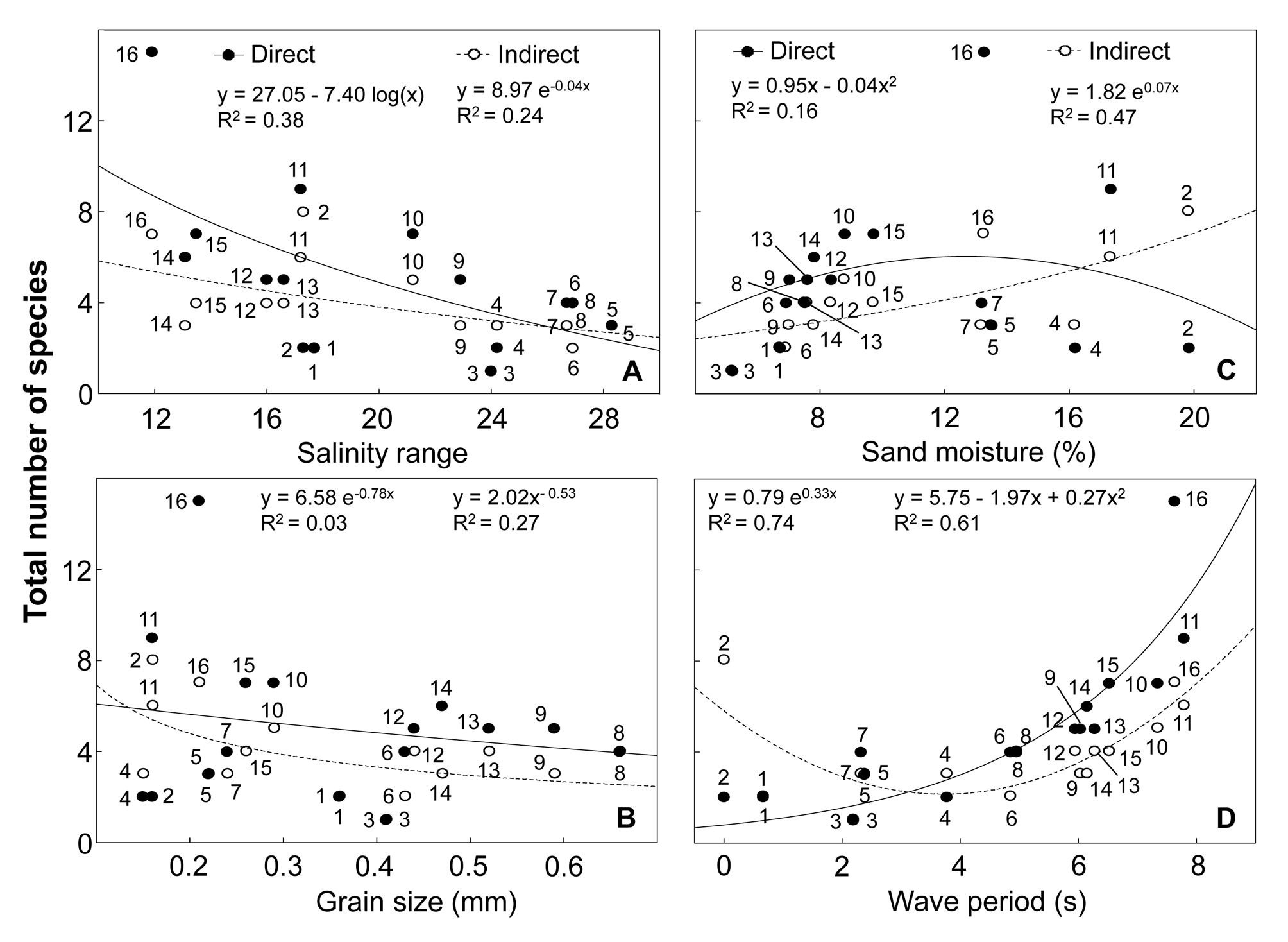

Supplement: Figure S4 — Relationship between species richness discriminated by development mode and environmental variables. (A) salinity range, (B) grain size, (C) sand moisture and (D) wave period. Beaches are numbered following Figure 1. Statistical details of the models fitted are presented in Table S3. (TIF) [file pone.0040468.s004.tif]

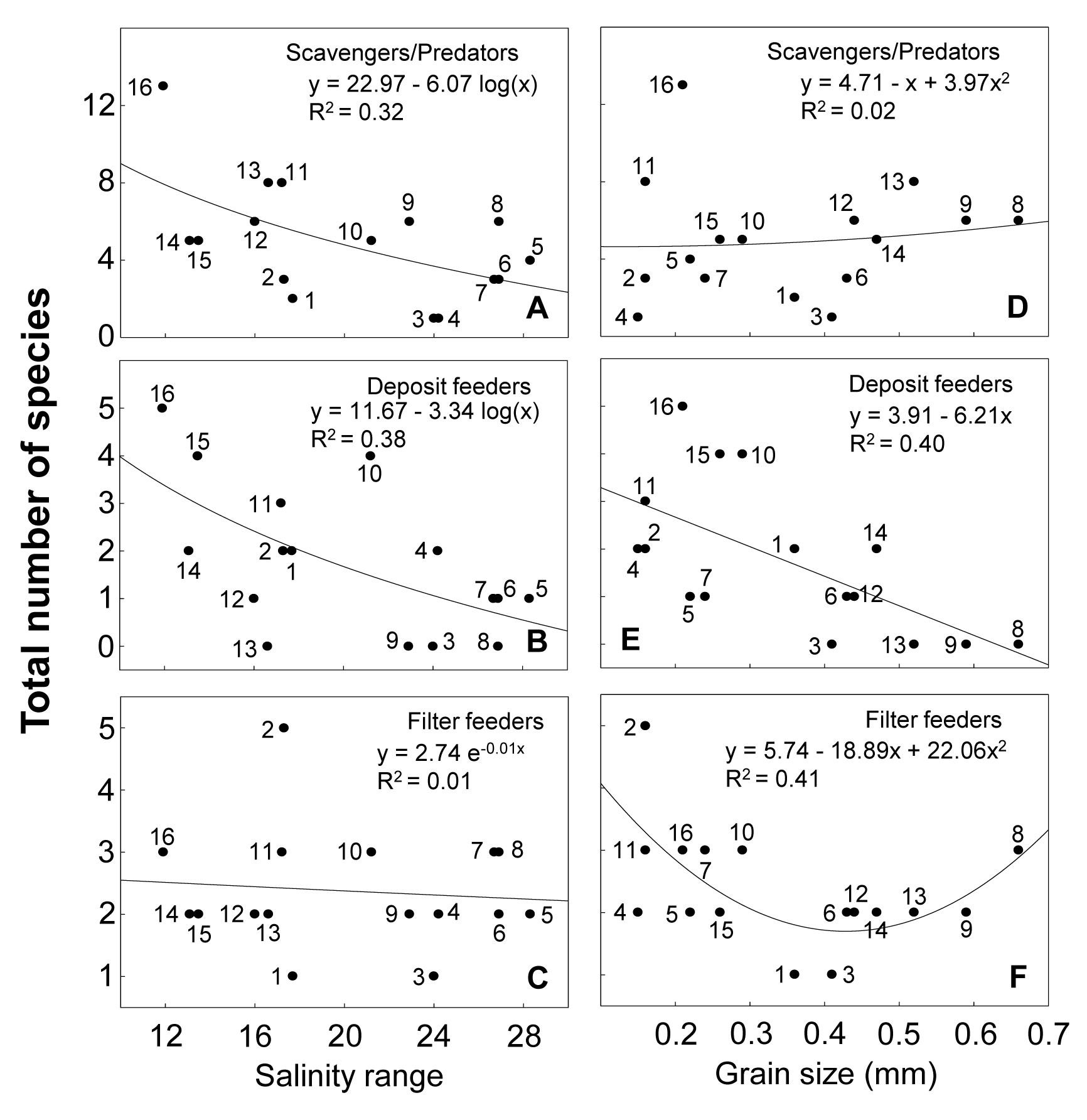

Supplement: Figure S5 — Relationship between species richness discriminated by feeding mode and salinity range and grain size. Beaches are numbered following Figure 1. Statistical details of the models fitted are presented in Table S3. (TIF) [file pone.0040468.s005.tif]

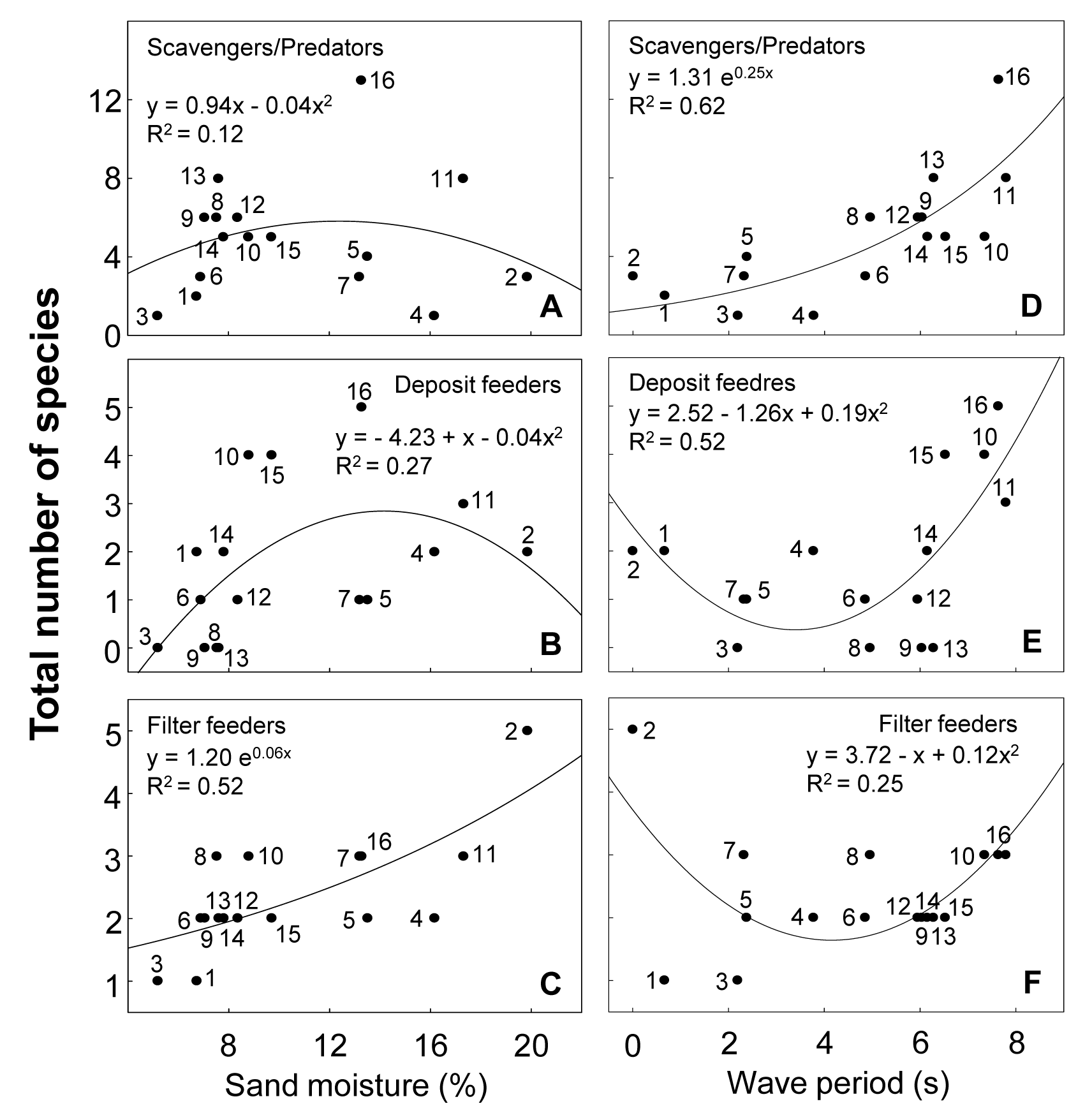

Supplement: Figure S6 — Relationship between species richness discriminated by feeding mode and sand moisture and wave period. Beaches are numbered following Figure 1. Statistical details of the models fitted are presented in Table S3. (TIF) [file pone.0040468.s006.tif]
