# Supplementary material for: Disentangling Diversity Patterns in Sandy Beaches along Environmental Gradients
Source: PLoS One. 2012 Jul 6;7(7):e40468. doi: 10.1371/journal.pone.0040468 (PMC3391285; doi:10.1371/journal.pone.0040468)
Supplement: Table S1 — Best models relating species richness of the whole community and environmental variables. ***p<0.001. (DOC) [file pone.0040468.s007.doc]

**Table S1. Models for integrated information.**

| Variable | Model | a | b | c | R^2^ |
| --- | --- | --- | --- | --- | --- |
| Salinity | y = a - x + cx^2^ | 14.22 |  | 0.04 | 0.47*** |
| Salinity range | y = ae^-bx^ | 48.55 | 0.06 |  | 0.41*** |
| Slope (%) | y = ae^-bx^ | 25.59 | 0.11 |  | 0.19*** |
| Grain size (mm) | y = ae^-x^ | 21.02 |  |  | 0.15*** |
| Swash width (m) | y = a - bx + cx^2^ | 18.78 | 3.96 | 0.39 | 0.69*** |
| Sand moisture (%) | y = bx - cx^2^ |  | 2.28 | 0.07 | 0.28*** |
| Wave period (s) | y = a - bx + cx^2^ | 15.75 | 5.22 | 0.85 | 0.72*** |
| Dean’s parameter Ω | y = ae^bx^ | 5.77 | 0.28 |  | 0.77*** |

Best models relating species richness of the whole community and environmental variables. ***p<0.001.
