# Supplementary material for: Disentangling Diversity Patterns in Sandy Beaches along Environmental Gradients
Source: PLoS One. 2012 Jul 6;7(7):e40468. doi: 10.1371/journal.pone.0040468 (PMC3391285; doi:10.1371/journal.pone.0040468)
Supplement: Table S2 — Fixed effects of the Generalized Linear Mixed Models relating species richness and environmental predictors. Model components are expressed in R language. All terms were significant (p<0.05), with the exception of intercept and poly (Temperature, 2)1. SE: Standard Error. (DOC) [file pone.0040468.s008.doc]

**Table S2. GLMM model.**

| Model Component | Estimate | SE | z value |
| --- | --- | --- | --- |
| Intercept | -0.333088 | 0.3316 | -1.005 |
| Salinity | 0.029804 | 0.0063 | 4.756 |
| Sand compaction | 0.242914 | 0.0775 | 3.135 |
| Wave period | 0.050759 | 0.0172 | 2.959 |
| poly (Temperature, 2)1 | 1.017625 | 0.5906 | 1.723 |
| poly (Temperature, 2)2 | -1.266413 | 0.5552 | -2.281 |

Fixed effects of the GLMM. Model components are expressed in R language. All terms were significant (p<0.05), with the exception of intercept and poly (Temperature, 2)1. SE: Standard Error.
