# Supplementary material for: Disentangling Diversity Patterns in Sandy Beaches along Environmental Gradients
Source: PLoS One. 2012 Jul 6;7(7):e40468. doi: 10.1371/journal.pone.0040468 (PMC3391285; doi:10.1371/journal.pone.0040468)
Supplement: Table S3 — Best models fitted for each deconstruction criterion based on taxonomic affiliation, beach zone occupied, development mode and feeding mode. **p<0.01, ***p<0.001, n.s.: non-significant. (DOC) [file pone.0040468.s009.doc]

**Table S3. Models for deconstruction based on taxonomic affiliation, beach zone occupied, development mode and feeding mode.**

| Deconstruction criterion | | Faunal category | | Variable | | Model | | a | b | c | R^2^ |
| --- | --- | --- | --- | --- | --- | --- | --- | --- | --- | --- | --- |
| Taxonomic affiliation | | Crustaceans | |  |  | |  |  |  |  |  |
|  |  |  |  | Salinity | | y = ae^bx^ | | 1.34 | 0.06 |  | 0.62*** |
|  |  |  |  | Salinity range | | y = ae^-bx^ | | 17.11 | 0.06 |  | 0.39*** |
|  |  |  |  | Slope (%) | | y = a - log(x) | | 7.19 |  |  | 0.05 n.s. |
|  |  |  |  | Grain size(mm) | | y = ae^-x^ | | 7.83 |  |  | 0.05 n.s. |
|  |  |  |  | Swash width (m) | | y = ae^bx^ | | 1.43 | 0.16 |  | 0.73*** |
|  |  |  |  | Sand moisture (%) | | y = bx - cx^2^ | |  | 1.01 | 0.04 | 0.19*** |
|  |  |  |  | Wave period (s) | | y = ae^bx^ | | 1.97 | 0.20 |  | 0.73*** |
|  |  |  |  | Dean’s parameter Ω | | y = ae^bx^ | | 2.51 | 0.24 |  | 0.70*** |
|  |  | Molluscs | |  | |  | |  |  |  |  |
|  |  |  |  | Salinity | | y = a - bx + cx^2^ | | 5.96 | 0.52 | 0.01 | 0.27*** |
|  |  |  |  | Salinity range | | y = ae^-bx^ | | 5.34 | 0.05 |  | 0.12** |
|  |  |  |  | Slope (%) | | y = ae^-bx^ | | 10.08 | 0.37 |  | 0.65*** |
|  |  |  |  | Grain size(mm) | | y = ae^-bx^ | | 7.73 | 4.65 |  | 0.42*** |
|  |  |  |  | Swash width (m) | | y = a - bx + cx^2^ | | 6.71 | 1.59 | 0.11 | 0.60*** |
|  |  |  |  | Sand moisture (%) | | y = ae^bx^ | | 0.44 | 0.13 |  | 0.63*** |
|  |  |  |  | Wave period (s) | | y = a - bx + cx^2^ | | 4.74 | 2.04 | 0.25 | 0.61*** |
|  |  |  |  | Dean’s parameter Ω | | y = ae^bx^ | | 0.48 | 0.36 |  | 0.65*** |
|  |  | Polychaetes | |  | |  | |  |  |  |  |
|  |  |  |  | Salinity | | y = a - bx + cx^2^ | | 5.29 | 0.61 | 0.02 | 0.41*** |
|  |  |  |  | Salinity range | | y = a - blog(x) | | 14.67 | 4.16 |  | 0.38*** |
|  |  |  |  | Slope (%) | | y = a - x + cx^2^ | | 5.39 |  | 0.07 | 0.20*** |
|  |  |  |  | Grain size(mm) | | y = a - bx + cx^2^ | | 5.74 | 18.39 | 20.45 | 0.12** |
|  |  |  |  | Swash width (m) | | y = a - x + cx^2^ | | 4.11 |  | 0.09 | 0.44*** |
|  |  |  |  | Sand moisture (%) | | y = - a + x - cx^2^ | | 3.94 |  | 0.03 | 0.15*** |
|  |  |  |  | Wave period (s) | | y = a - bx + cx^2^ | | 3.68 | 1.85 | 0.26 | 0.64*** |
|  |  |  |  | Dean’s parameter Ω | | y = e^bx^ | |  | 0.26 |  | 0.41*** |
|  |  | Insects | |  | |  | |  |  |  |  |
|  |  |  |  | Salinity | | y = e^bx^ | |  | 0.07 |  | 0.62*** |
|  |  |  |  | Salinity range | | y = a - bx + cx^2^ | | 30.61 | 2.50 | 0.06 | 0.45*** |
|  |  |  |  | Slope (%) | | y = a + x - cx^2^ | | 3.38 |  | 0.12 | 0.08 n.s. |
|  |  |  |  | Grain size(mm) | | y = a - x - cx^2^ | | 5.84 |  | 5.12 | 0.10 n.s. |
|  |  |  |  | Swash width (m) | | y = ae^bx^ | | 1.10 | 0.17 |  | 0.70*** |
|  |  |  |  | Sand moisture (%) | | y = - a + bx - cx^2^ | | 10.86 | 2.85 | 0.11 | 0.54*** |
|  |  |  |  | Wave period (s) | | y = ae^bx^ | | 1.67 | 0.20 |  | 0.58*** |
|  |  |  |  | Dean’s parameter Ω | | y = ae^bx^ | | 2.07 | 0.26 |  | 0.70*** |
| Beach zone occupied | | Intertidal | |  |  | |  |  |  |  |  |
|  |  |  |  | Salinity | | y = a - bx + cx^2^ | | 16.95 | 1.64 | 0.05 | 0.39*** |
|  |  |  |  | Salinity range | | y = ae^-bx^ | | 33.56 | 0.07 |  | 0.41*** |
|  |  |  |  | Slope (%) | | y = ae^-bx^ | | 21.22 | 0.19 |  | 0.38*** |
|  |  |  |  | Grain size(mm) | | y = ax^-b^ | | 3.79 | 0.65 |  | 0.28*** |
|  |  |  |  | Swash width (m) | | y = a - bx + cx^2^ | | 17.31 | 4.10 | 0.33 | 0.65*** |
|  |  |  |  | Sand moisture (%) | | y = ae^bx^ | | 3.89 | 0.07 |  | 0.34*** |
|  |  |  |  | Wave period (s) | | y = a - bx + cx^2^ | | 12.94 | 5.51 | 0.77 | 0.76*** |
|  |  |  |  | Dean’s parameter Ω | | y = ae^bx^ | | 2.49 | 0.33 |  | 0.77*** |
|  |  | Supralittoral | |  |  | |  |  |  |  |  |
|  |  |  |  | Salinity | | y = ae^bx^ | | 1.37 | 0.07 |  | 0.73*** |
|  |  |  |  | Salinity range | | y = a - bx + cx^2^ | | 40.47 | 3.34 | 0.08 | 0.43*** |
|  |  |  |  | Slope (%) | | y = a + x - cx^2^ | | 4.56 |  | 0.11 | 0.01 n.s. |
|  |  |  |  | Grain size(mm) | | y = a + x - cx^2^ | | 6.58 |  | 2.96 | 0.01 n.s. |
|  |  |  |  | Swash width (m) | | y = ae^bx^ | | 1.70 | 0.16 |  | 0.74*** |
|  |  |  |  | Sand moisture (%) | | y = - a + bx - cx^2^ | | 12.91 | 3.68 | 0.15 | 0.56*** |
|  |  |  |  | Wave period (s) | | y = ae^bx^ | | 2.63 | 0.18 |  | 0.60*** |
|  |  |  |  | Dean’s parameter Ω | | y = ae^bx^ | | 3.37 | 0.21 |  | 0.54*** |
| Development mode | | Direct | |  |  | |  |  |  |  |  |
|  |  |  |  | Salinity | | y = e^bx^ | |  | 0.07 |  | 0.57*** |
|  |  |  |  | Salinity range | | y = a - blog(x) | | 27.05 | 7.40 |  | 0.38*** |
|  |  |  |  | Slope (%) | | y = a + x - cx^2^ | | 3.83 |  | 0.13 | 0.05 n.s. |
|  |  |  |  | Grain size(mm) | | y = ae^-bx^ | | 6.58 | 0.78 |  | 0.03 n.s. |
|  |  |  |  | Swash width (m) | | y = ae^bx^ | | 0.56 | 0.25 |  | 0.79*** |
|  |  |  |  | Sand moisture (%) | | y = bx - cx^2^ | |  | 0.95 | 0.04 | 0.16** |
|  |  |  |  | Wave period (s) | | y = ae^bx^ | | 0.79 | 0.33 |  | 0.74*** |
|  |  |  |  | Dean’s parameter Ω | | y = ae^bx^ | | 1.51 | 0.35 |  | 0.76*** |
|  |  | Indirect | |  |  | |  |  |  |  |  |
|  |  |  |  | Salinity | | y = a - x + cx^2^ | | 10.09 |  | 0.03 | 0.17** |
|  |  |  |  | Salinity range | | y = ae^-bx^ | | 8.97 | 0.04 |  | 0.24*** |
|  |  |  |  | Slope (%) | | y = ax^-b^ | | 10.14 | 0.65 |  | 0.52*** |
|  |  |  |  | Grain size(mm) | | y = ax^-b^ | | 2.02 | 0.53 |  | 0.27*** |
|  |  |  |  | Swash width (m) | | y = a - bx + cx^2^ | | 7.67 | 1.56 | 0.12 | 0.56*** |
|  |  |  |  | Sand moisture (%) | | y = ae^bx^ | | 1.82 | 0.07 |  | 0.47*** |
|  |  |  |  | Wave period (s) | | y =a - bx + cx^2^ | | 5.75 | 1.97 | 0.27 | 0.61*** |
|  |  |  |  | Dean’s parameter Ω | | y = ae^bx^ | | 1.76 | 0.21 |  | 0.62*** |
| Feeding mode | | Scavengers/Predators | |  |  | |  |  |  |  |  |
|  |  |  |  | Salinity | | y = e^bx^ | |  | 0.07 |  | 0.57*** |
|  |  |  |  | Salinity range | | y = a - blog(x) | | 22.97 | 6.07 |  | 0.32** |
|  |  |  |  | Slope (%) | | y = a - x + cx^2^ | | 7.31 |  | 0.09 | 0.03 n.s. |
|  |  |  |  | Grain size(mm) | | y = a - x + cx^2^ | | 4.71 |  | 3.97 | 0.02 n.s. |
|  |  |  |  | Swash width (m) | | y = ae^bx^ | | 0.93 | 0.19 |  | 0.58*** |
|  |  |  |  | Sand moisture (%) | | y = bx - cx^2^ | |  | 0.94 | 0.04 | 0.12** |
|  |  |  |  | Wave period (s) | | y = ae^bx^ | | 1.31 | 0.25 |  | 0.62*** |
|  |  |  |  | Dean’s parameter Ω | | y = ae^bx^ | | 2.02 | 0.27 |  | 0.40*** |
|  |  | Deposit feeders | |  |  | |  |  |  |  |  |
|  |  |  |  | Salinity | | y = a - bx + cx^2^ | | 4.07 | 0.46 | 0.01 | 0.31*** |
|  |  |  |  | Salinity range | | y = a - blog(x) | | 11.67 | 3.34 |  | 0.38*** |
|  |  |  |  | Slope (%) | | y = a - blog(x) | | 4.51 | 1.77 |  | 0.27*** |
|  |  |  |  | Grain size(mm) | | y = a - bx | | 3.91 | 6.21 |  | 0.40*** |
|  |  |  |  | Swash width (m) | | y = a - x + cx^2^ | | 3.41 |  | 0.09 | 0.61*** |
|  |  |  |  | Sand moisture (%) | | y = - a + x - cx^2^ | | 4.23 |  | 0.04 | 0.27*** |
|  |  |  |  | Wave period (s) | | y = a - bx + cx^2^ | | 2.52 | 1.26 | 0.19 | 0.52*** |
|  |  |  |  | Dean’s parameter Ω | | y = ae^bx^ | | 0.42 | 0.41 |  | 0.66*** |
|  |  | Filter feeders | |  |  | |  |  |  |  |  |
|  |  |  |  | Salinity | | y = a - bx + cx^2^ | | 3.32 | 0.14 | 0.004 | 0.06 n.s. |
|  |  |  |  | Salinity range | | y = ae^-bx^ | | 2.74 | 0.01 |  | 0.01 n.s. |
|  |  |  |  | Slope (%) | | y = a - bx + cx^2^ | | 6.95 | 1.70 | 0.14 | 0.69*** |
|  |  |  |  | Grain size(mm) | | y = a - bx + cx^2^ | | 5.74 | 18.89 | 22.06 | 0.41*** |
|  |  |  |  | Swash width (m) | | y = a - bx + cx^2^ | | 4.22 | 0.66 | 0.05 | 0.31*** |
|  |  |  |  | Sand moisture (%) | | y = ae^bx^ | | 1.20 | 0.06 |  | 0.52*** |
|  |  |  |  | Wave period (s) | | y = a - x + cx^2^ | | 3.72 |  | 0.12 | 0.25*** |
|  |  |  |  | Dean’s parameter Ω | | y = ae^bx^ | | 1.50 | 0.12 |  | 0.36*** |

Best linear and non-linear models relating species richness of faunal categories and environmental variables. **p<0.01, ***p<0.001, n.s.: non-significant.
