# Supplementary material for: Disentangling Diversity Patterns in Sandy Beaches along Environmental Gradients
Source: PLoS One. 2012 Jul 6;7(7):e40468. doi: 10.1371/journal.pone.0040468 (PMC3391285; doi:10.1371/journal.pone.0040468)
Supplement: Table S4 — Best models relating abundance of selected species with grain size and salinity. **p<0.01, ***p<0.001. (DOC) [file pone.0040468.s010.doc]

**Table S4. Models for deconstruction by species.**

| Variable | | Species | | Model | | a | b | R^2^ |
| --- | --- | --- | --- | --- | --- | --- | --- | --- |
| Grain size (mm) | |  |  | |  |  |  |  |
|  |  | *Excirolana armata* | | y = ae^-bx^ | | 12952.99 | 4.83 | 0.27** |
|  |  | *Atlantorchestoidea brasiliensis* | | y = ax^b^ | | 5565.91 | 2.68 | 0.77*** |
| Salinity |  |  | |  | |  |  |  |
|  |  | *Erodona mactroides* | | y = ae^-bx^ | | 43190.56 | 0.22 | 0.66*** |
|  |  | *Donax haleyanus* | | y = ae^bx^ | | 0.00003 | 0.59 | 0.37** |

Best models relating abundance of selected species with grain size and salinity. **p<0.01, ***p<0.001.
